# On Divided-Type Connectivity of Graphs

**DOI:** 10.3390/e25010176

**Published:** 2023-01-16

**Authors:** Qiao Zhou, Xiaomin Wang, Bing Yao

**Affiliations:** 1 College of Information and Electrical Engineering, China Agricultural University, Beijing 100083, China; 2 Beijing Institute of Remote Sensing Equipment, Beijing 100854, China; 3 College of Mathematics and Statistics, Northwest Normal University, Lanzhou 730070, China; 4 School of Electronics and Information Engineering, Lanzhou Jiaotong University, Lanzhou 730070, China

**Keywords:** divided operation, coincident operation, divided connectivity, Euler graph

## Abstract

The graph connectivity is a fundamental concept in graph theory. In particular, it plays a vital role in applications related to the modern interconnection graphs, e.g., it can be used to measure the vulnerability of the corresponding graph, and is an important metric for reliability and fault tolerance of the graph. Here, firstly, we introduce two types of divided operations, named *vertex-divided operation* and *edge-divided operation*, respectively, as well as their inverse operations *vertex-coincident operation* and *edge-coincident operation*, to find some methods for splitting vertices of graphs. Secondly, we define a new connectivity, which can be referred to as divided connectivity, which differs from traditional connectivity, and present an equivalence relationship between traditional connectivity and our divided connectivity. Afterwards, we explore the structures of graphs based on the vertex-divided connectivity. Then, as an application of our divided operations, we show some necessary and sufficient conditions for a graph to be an Euler’s graph. Finally, we propose some valuable and meaningful problems for further research.

## 1. Introduction and Researching Background

Graph connectivity is one of the most basic concepts used in the application of graph theory, both in the combinatorial sense and in the algorithmic sense. Especially, it plays an important role in applications related to graph embedding. The connectivity can serve to assess the vulnerability of the corresponding graph and measure the capability of connection for a set of vertices in the graph. To better understand the characteristics of graph connectivity, a wide range of technical methods were developed and then used to analyze various problems.

**Figure 1:**
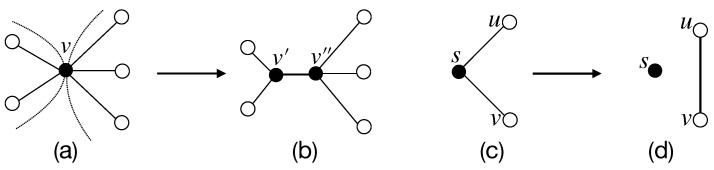
A scheme for illustrating vertex-splitting operation and edge-splitting operation: vertex-splitting operation is from (**a**) to (**b**); edge-splitting operation is from (**c**) to (**d**).

This classical issue has attracted attention to understanding and utilizing various operations regarding graphs. By consulting the literature, we found that the splitting operations on graphs can be divided two classes: one is the vertex-splitting operation and another is the edge-splitting operation. Figure 1 explains the vertex-splitting process and the edge-splitting process. The former operation can be defined as follows: “A vertex *v* of degree i=deg(v) is splitted into two new vertices $v^{\prime }$ and $v^{\prime \prime }$ with degrees $k=deg(v^{\prime })$ and $l=deg(v^{\prime \prime })=i+2-k$ by adding a new edge to join $v^{\prime }$ and $v^{\prime \prime }$ together”. As several examples, Cheah et al. obtained an $O(n^{3})$ algorithm for recognizing a trapezoid graph [1]. Mertzios et al. presented a new method of augmenting a given graph and used vertex-splitting in a trapezoid graph [2]. Hilton et al. studied graphs which are critical with respect to the chromatic index [3], and so forth. The latter operation can be explained as follows: “in an undirected graph, splitting off two edges incident to a vertex *s*, say (s,u) and (s,v), means deleting them and adding a new edge (u,v)”, mainly applied to solve connectivity problems. For example, Nagamochi presented several algorithms for splitting all edges connect to a vertex *s* of even degree in a graph *G* with *n* vertices and *m* edges, namely, $O\left(nm\log n+n^{2}\log^{2}n\right)=\tilde{O}\left(nm\right)$ for a graph [4], $O(n^{3}\log n)$ for planar graph [5,6], and $O(mn+n^{2}\log n)$ for edge-weighted graphs [7]. Fukunaga and Nagamochi presented if and only if for a given graph/digraph to have an Eulerian detachment that satisfies a given local edge-connectivity requirement [8]. Farooq et al. described experimental implementations of graph splitting at vertices and edge cutting [9,10].

Although the aforementioned two operations can be used to solve some problems, these two operations cannot be applied to solve the issue that a vertex be divided into multiple vertices, nor can they be used to solve problems where the splitting vertices synthesize a vertex. Here, we introduce two types of divided operations, called *v-divided operation* and *e-divided operation*, respectively, and their inverse operations, *v-coincident operation and e-coincident operation*, as we will show shortly.

Since many graphs in the current real world are weighted, and they are composed of small block (modular) graphs, graphs just organically combine them into a whole, which is also the most natural and reasonable technique.By splitting and refining the network, the minimal structural features are obtained. Similar to how matter is made up of molecules, ions and atoms, the minimal structural features of networks can help us to understand the structure and topological properties of graphs. Battaglia et al., in [11], points out: “*It is unclear the best ways to convert sensory data into more structured representations like graphs*”. Our divided operation preserves the “molecules, ions and atoms” of the original weighted network, which is conductive to reconstructing the original weighted network in polynomial time without the need of “*requiring the ability to add or remove edges depending on context*”. Because our divided connectivity is equivalent to the traditional connectivity, the reliability of our divided connectivity is proven.

The remaining sections of our article are organized as follows. We present a preliminary introductionin Section 2, in which some terminology and notations are given, our divided operations are introduced, and two parameters of graphs regarding the divided connectivity are defined. In Section 3, we discuss the connections on various graph connectivities, present an equivalent relationship between traditional connectivity and our divided connectivity, and show the topological structures of graphs by our divided technique. As an application of our divided operations, we show some necessary and sufficient conditions for a graph to be an Euler’s graph. An elaborate conclusion summarizes the above works and proposes possible problems for further investigation of various connectivities in the last section.

## 2. Divided Operations

The following operations on graphs are discussed in this article. For distinction, we will use “divide” or “divided” in our definitions instead of “split” or “splitting”, since our operations differ from “edge-splitting” and “vertex-splitting” used in the existing published articles. A simple graph is one having no multiple-edge and self-edge. Let N(x) be the set of all neighbors of a vertex *x* in a simple graph, and we call N(x)*neighbor set*, so the cardinality |N(x)| is defined as the degree of the vertex *x*. We present two types of divided operations [12]. The mathematical symbols apllied in our paper are shown in Table 1.

- **Vertex-divided operation and vertex-coincident operation.** For the neighbor set $N\left(x\right)=\{u_{i}:i\in \left[1,n\right]\}$ of a vertex *x* of a simple graph *G*, where *n* is the degree of *x*, we define a *vertex-divided operation* (v-divided operation) to *x* as follows: Divide *x* into two vertices $x_{1},x_{2}$, and then join $x_{1}$ with vertices $u_{1},u_{2},\ldots ,u_{i}$ with respect to n>i≥1, and then join $x_{2}$ with vertices $u_{i+1},\ldots ,u_{n}$ for n−i≥1; finally, the resultant graph is denoted as G∧x. If two neighbor sets N(x) and N(y) of two vertices x,y of a simple graph *G* hold N(x)∩N(y)=∅ true, we coincide *x* with *y* into one vertex w=x∘y such that N(w)=N(x)∪N(y), and refer to this procedure as a *vertex-coincident operation* (v-coincident operation); the resultant graph is denoted as G(x∘y).
- **Edge-divided operation and edge-coincident operation.** Let uv be an edge of a simple graph *G* with the neighbor sets $N\left(u\right)=\{x_{s}:s\in \left[1,j\right]\}$ and $N\left(v\right)=\{y_{t}:t\in \left[1,n\right]\}$. We divide the edge uv into two edges $u^{\prime }v^{\prime }$ and $u^{\prime \prime }v^{\prime \prime }$ such that $N\left(u^{\prime }\right)=\left\{x_{s}:s\in \left[1,i\right]\right\}$ and $N\left(u^{\prime \prime }\right)=\left\{x_{s}:s\in \left[i+1,j\right]\right\}$, holding j−i≥1 true, as well as $N\left(v^{\prime }\right)=\left\{y_{t}:t\in \left[1,k\right]\right\}$ and $N\left(v^{\prime \prime }\right)=\left\{y_{t}:t\in \left[k+1,n\right]\right\}$, holding n−k≥1 true, and the resultant graph is denoted as G∧uv; this procedure is called an *edge-divided operation* (e-divided operation). Conversely, we coincide two edges $u^{\prime }v^{\prime }$ and $u^{\prime \prime }v^{\prime \prime }$ of the graph H=G∧uv into one, and the resultant graph is written as $H(u^{\prime }v^{\prime }⊖u^{\prime \prime }v^{\prime \prime })$ if $N\left(u^{\prime }\right)\cap N\left(u^{\prime \prime }\right)=\emptyset$ and $N\left(v^{\prime }\right)\cap N\left(v^{\prime \prime }\right)=\emptyset$; we name the procedure of obtaining $H(u^{\prime }v^{\prime }⊖u^{\prime \prime }v^{\prime \prime })$ as *edge-coincident operation* (e-coincident operation).

**Figure 2:**
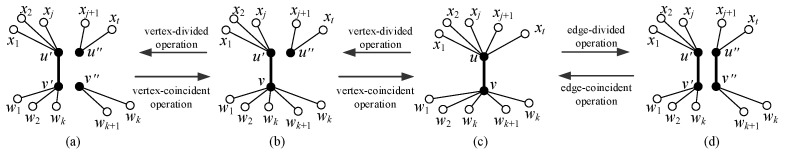
A schemefor illustrating four graph operations: (**a**) v-divided operation; (**b**) v-coincident operation; (**c**) e-divided operation; and (**d**) e-coincident operation, cited from [12].

In Figure 2, a v-divided operation is from (c) to (b), and another v-divided operation is from (b) to (a); a v-coincident operation is from (a) to (b), and another v-coincident operation is from (b) to (c). An *e-divided operation* is just from (c) to (d); and an *e-coincident operation* is from (d) to (c). In Figure 2, after a group of divided operations, then the neighbor sets hold $N\left(u^{\prime }\right)\cap N\left(u^{\prime \prime }\right)=\emptyset$ and $N\left(v^{\prime }\right)\cap N\left(v^{\prime \prime }\right)=\emptyset$ in the resultant graph. We perform a v-divided operation to a vertex *u* of a simple graph *H*, so the vertex set satisfies |V(H∧u)|=1+|V(H)| and the edge set holds |E(H∧u)|=|E(H)| (see Figure 2b). The resultant graph obtained by performing an e-divided operation to an edge uv of *H* holds |V(H∧uv)|=2+|V(H)| and |E(H∧uv)|=1+|E(H)| true (see Figure 2d).

**Remark** **1.**

(1) *Let f be an attribute of a network N(t) at time step t, the evaluation f(x,t) of each vertex x is called* vertex weight, *and the evaluation f(uv,t) of each edge uv is called* edge weight. *Thus, we say that N(t) is a* weighted network. *For example, we have $f\left(u,t\right)=f\left(u^{\prime },t\right)+f\left(u^{\prime \prime },t\right)$ and $f\left(v,t\right)=f\left(v^{\prime },t\right)+f\left(v^{\prime \prime },t\right)$ in Figure 2a–c; and $f\left(uv,t\right)=f\left(u^{\prime }v^{\prime },t\right)+f\left(u^{\prime \prime }v^{\prime \prime },t\right)$ in Figure 2c,d, respectively. Thereby, the v-divided graph N(t)∧u and the e-divided graph N(t)∧uv keep the complete weighted information of the original network N(t).*
(2) *The resultant graph obtained by deleting a vertex x from a simple graph G is denoted as G−x (v-deleted), and deleting an edge xy from the graph produces a simple graph denoted as G−xy (e-deleted). Clearly, the v-deleted (respectively, e-deleted) graph G−x (respectively, G−xy) is unique, but the v-divided (respectively, e-divided) graph G∧x (respectively, G∧xy) is not unique, in general. However, it is difficult to reconstruct the original graph G from the v-deleted (respectively, e-deleted) graph G−x (respectively, G−xy), although it is easy for the v-divided (respectively, e-divided) graph G∧x (respectively, G∧xy), because G∧x (respectively, G∧xy) maintains the complete structure information of the original graph G.*
(3) *The vertex deletion technique is applied to many issues in mathematics, such as the famous Kelly–Ulam’s reconstruction conjecture proposed in 1942: Let both G and H be graphs with n vertices. If there is a bijection f:V(G)→V(H) such that two vertices deleted graphs G−u≅H−f(u) for each vertex u∈V(G), then these two graphs G and H are isomorphic to each other, that is, G≅H [13]. However, we claim that G≅H if G∧u≅H∧f(u) for each vertex u∈V(G).*

**Figure 3:**
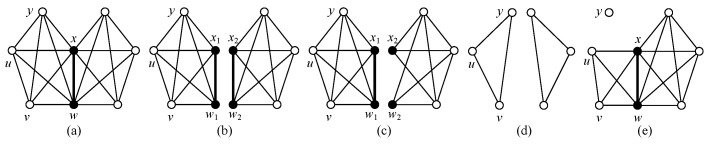
(**a**) A graph *H* with minimum degree δ(H)=4; (**b**) an e-divided graph H∧xw with $\kappa_{d}^{\prime }\left(H\right)=1$; (**c**) a v-divided graph H∧{x,w} with $\kappa_{d}\left(H\right)=2$; (**d**) a v-deleted graph H−{x,w} with κ(H)=2; (**e**) an e-deleted graph H−{yx,yw,yu,yv} with $\kappa^{\prime }\left(H\right)=4$.

We show two parameters of graphs based on the divided connectivity:

**The v-divided connectivity.** A *v-divided k-connected graph H* holds: $H\wedge V^{*}$ (or $H\wedge {\left\{x_{i}\right\}}_{1}^{k}$) is disconnected, where $V^{*}=\left\{x_{1},x_{2},\ldots ,x_{k}\right\}$ is a subset of V(H), each component $H_{j}$ of $H\wedge {\left\{x_{i}\right\}}_{1}^{k}$ has at least a vertex $w_{j}\notin V^{*}$, $|V\left(H\wedge {\left\{x_{i}\right\}}_{1}^{k}\right)|=k+|V\left(H\right)|$ and $|E\left(H\wedge {\left\{x_{i}\right\}}_{1}^{k}\right)|=|E\left(H\right)|$. The smallest number of *k* for which $H\wedge {\left\{x_{i}\right\}}_{1}^{k}$ is disconnected is called the *v-divided connectivity* of *H*, denoted as $\kappa_{d}\left(H\right)$ (see example shown in Figure 3).

**The e-divided connectivity.** An *e-divided k-connected graph H* holds: $H\wedge {\left\{e_{i}\right\}}_{1}^{k}$ (or $H\wedge E^{*}$) is disconnected, where $E^{*}=\left\{e_{1},e_{2},\ldots ,e_{k}\right\}$ is a subset of E(H), each component $H_{j}$ of $H\wedge {\left\{e_{i}\right\}}_{1}^{k}$ has at least a vertex $w_{j}$ being not any end of any edge of $E^{*}$, $|V\left(H\wedge {\left\{e_{i}\right\}}_{1}^{k}\right)|=2k+|V\left(H\right)|$ and $|E\left(H\wedge {\left\{e_{i}\right\}}_{1}^{k}\right)|=k+|E\left(H\right)|$. The smallest number of *k* for which $H\wedge {\left\{e_{i}\right\}}_{1}^{k}$ is disconnected is called the *e-divided connectivity* of *H*, denoted as $\kappa_{d}^{\prime }\left(H\right)$ (see example shown in Figure 3).

Recall that the minimum degree δ(H), the vertex connectivity κ(H), and the edge connectivity $\kappa^{\prime }\left(H\right)$ of a simple graph *G* hold the following inequalities [13] true:

(1) $$\kappa \left(H\right)\leq \kappa^{\prime }\left(H\right)\leq \delta \left(H\right)$$

However, we do not have the inequalities (1) about the minimum degree δ(H), the v-divided connectivity $\kappa_{d}\left(H\right)$, and the e-divided connectivity $\kappa_{d}^{\prime }\left(H\right)$ for a simple graph *H*.

## 3. Some Connections between Graph Connectivities

### 3.1. Connection between Traditional Connectivity and Divided Connectivity

**Lemma** **1.** *A graph G is k-connected if and only if it is v-divided k-connected, namely, $\kappa_{d}\left(H\right)=\kappa \left(H\right)$.*

**Proof.**  *The proof of “if”.* Suppose that *G* is a *k*-connected graph, and G−S is disconnected with S⊂V(G) and |S|=k. Let $G_{1},G_{2},\ldots ,G_{m}$ be the components of the disconnected graph G−S. Apparently,

(1) m≥2, it is evident.
(2) Each vertex x∈S must be adjacent with some vertex $u_{x,i}\in V\left(G_{i}\right)$ for each i=1,2,…,m, otherwise, there is a proper subset $S^{*}\subset S$ with $|S^{*}\left|<|S\right|$, such that $G-S^{*}$ is disconnected immediately: a contradiction.
(3) By the above (2), we have *m* subgraphs $H_{i}$ of *G* induced by sets $S\cup V(G_{i})$ with i=1,2,…,m. We call $H_{i}$ a *block* of *G*. Thereby, we have that $V\left(H_{i}\right)\cap V\left(H_{j}\right)=S$ for i≠j and ${⋂}_{i=1}^{m}V\left(H_{i}\right)=S$, which shows that *G* is v-divided *k*-connected after performing the v-divided operations to the vertices of *S*, and the v-divided graph G∧S has subgraphs $H_{1},H_{2},\ldots ,H_{m}$.
(4) We have subgraphs $L_{1},L_{2},\ldots ,L_{n}$ of the v-divided graph G∧S with n≥2, where $L_{j}={⋃}_{i=1}^{m_{j}}H_{j,i}$ for j=1,2,…,n and $\sum_{j=1}^{n}m_{j}=m$, as well as $V\left(L_{s}\right)\cap V\left(L_{t}\right)=S$ for s≠t.
(5) If *G* is v-divided $k^{*}$-connected with $k^{*}<k$, then there exists a subset X⊂V(G) with $k^{*}=\left|X\right|$ such that the v-divided graph G∧X has subgraphs $R_{1},R_{2},\ldots ,R_{a}$ after performing a series of v-divided operations to the vertices of *X*, and $V\left(R_{i}\right)\cap V\left(R_{j}\right)=X$ for i≠j. Thereby, G−X is disconnected, and this contradicts the hypothesis of the proof of “if”.

*The proof of “only if”.* Suppose that *G* is a v-divided *k*-connected graph, that is, there exists a subset Y⊂V(G) with |Y|=k, such that the v-divided graph G∧Y has subgraphs $G_{1}^{\prime },G_{2}^{\prime },\ldots ,G_{b}^{\prime }$ holding $|V\left(G_{i}^{\prime }\right)\cap V\left(G_{j}^{\prime }\right)|=Y$ for i≠j. Thus, G−Y is a disconnected graph with components $G_{j}^{\prime }-Y$ for j=1,2,…,b, which means that *G* is *k*-connected. Conversely, if *G* is $k^{\prime }$-connected with $k^{\prime }<k$, then we can obtain that *G* is a v-divided $k^{\prime }$-connected graph by the proof of “if” above: it is an obvious conflict. We are finished. □

Lemma 1 enables us to obtain the subsequent result:

**Theorem** **1.** *If a k-connected graph has a property related with its k-connectivity, so does a v-divided k-connected graph.*

For example, Menger’s theorem (Karl Menger, 1927) states the following: “*Let G be a graph of order greater than k+1. Then G is k-connected if and only if any two distinct vertices of G are connected by at least k mutually internally-disjoint paths*”. Thus, each v-divided *k*-connected graph has at least *k* internally-disjoint paths to join any pair of vertices.

**Remark** **2.**

(1) *A k-connected graph G induces that the disconnected graph G−S has mutually-disjoint subgraphs $G_{1},G_{2},\ldots ,G_{m}$, where S is a subset of vertices of G and |S|=k. Evidently, these mutually-disjoint subgraphs $G_{1},G_{2},\ldots ,G_{m}$ are fixed. However, the v-divided graph G∧S may have its subgraphs $L_{1},L_{2},\ldots ,L_{n}$ with 2≤n≤m.*
(2) *We point out that the reconstruction of G from the v-divided graph G∧S is easier than that based on the vertex-deleting graph G−S. Recall Kelly–Ulam’s reconstruction conjecture (1942); unfortunately, this reconstruction conjecture is still open now.*

**Theorem** ** 2.** *Any connected graph G holds the inequalities $\kappa_{d}^{\prime }\left(G\right)\leq \kappa_{d}\left(G\right)\leq 2\kappa_{d}^{\prime }\left(G\right)$ true, and the boundaries are reachable.*

**Proof.**  First of all, $\kappa_{d}^{\prime }\left(K_{3}\right)=0$ and $\kappa_{d}^{\prime }\left(P_{3}\right)=0$. Let *G* be a connected graph being not $K_{3}$ and having the longest path $P_{a}$ with a≥4. Since *G* is a v-divided *k*-connected graph with $k=\kappa_{d}\left(G\right)$, it is *k*-connected too, by Lemma 1. There exists a subset S⊂V(G) with |S|=k such that G−S is a disconnected graph having components $G_{1},G_{2},\ldots ,G_{n}$. We construct subgraphs $H_{i}$ holding $V\left(H_{i}\right)=V\left(G_{i}\right)\cup S$ and $E\left(H_{i}\right)=E\left(G_{i}\right)\cup \left\{x_{i}y_{j}:\;x_{i}\in V\left(G_{i}\right),y_{j}\in S\right\}$. Notice that each vertex $y_{j}\in S$ is adjacent with some vertex of $G_{i}$ for i=1,2,…,n. Consequently, $H_{1},H_{2},\ldots ,H_{n}$ is just the v-divided graph G∧S.

**Figure 4:**
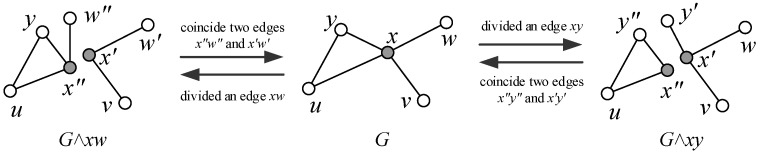
A scheme for illustrating the proof of Theorem 2.

If k=1, namely, S={w}, the v-divided graph G∧S has only $H_{1},H_{2}$ such that $V\left(H_{1}\right)\cap V\left(H_{2}\right)=\left\{w\right\}$. Without loss of generality, $H_{1}$ contains a path $P_{b}=wx_{1}x_{2}\ldots x_{b}$ with b≥2. Thus, we can divide the edge $wx_{1}$ of $G=H_{1}\cup H_{2}$ into two edges, $w^{\prime }x_{1}^{\prime }$ and $w^{\prime \prime }x_{1}^{\prime \prime }$, for obtaining two $H_{1}^{\prime },H_{2}^{\prime }$ such that $H_{1}^{\prime }=H_{1}$ with $w^{\prime }x_{1}^{\prime }=wx_{1}$, and $H_{2}^{\prime }=H_{2}+w^{\prime \prime }x_{1}^{\prime \prime }$, where $x_{1}^{\prime \prime }$ is a leaf of $H_{2}^{\prime }$, $w^{\prime \prime }=w$. Clearly, $|V\left(H_{1}^{\prime }\right)\setminus \left\{w,x_{1}\right\}|\geq 1$, so $G\wedge wx_{1}$ is an e-divided graph with $\kappa_{d}^{\prime }\left(G\right)=1$ (see Figure 4).

Considering the case k≥2, we can obtain two graphs $G_{1}^{*}$ and $G_{2}^{*}$ from $H_{1},H_{2},\ldots ,H_{n}$ of the v-divided graph G∧S by (4) of the proof of Lemma 1, such that $V\left(G_{1}^{*}\right)\cap V\left(G_{2}^{*}\right)=S$, so there are edges $x_{i}y_{i}$ of $G_{1}^{*}$ holding $x_{i}\in V\left(G_{1}^{*}\right)\setminus S$ and $y_{i}\in S=\left\{y_{1},y_{2},\ldots ,y_{k}\right\}$, such that $|V\left(G_{1}^{*}\right)\setminus \left\{x_{i},y_{i}\right\}|\geq 1$. Thereby, we divide each edge $x_{i}y_{i}$ into two $x_{i}^{\prime }y_{i}^{\prime }$ and $x_{i}^{\prime \prime }y_{i}^{\prime \prime }$ to obtain two graphs, $H_{1}^{*}$ and $H_{2}^{*}$, such that $H_{1}^{*}=G_{1}^{*}$ with $x_{i}^{\prime }y_{i}^{\prime }=x_{i}y_{i}$, $H_{2}^{*}=G_{2}^{*}+\left\{x_{i}^{\prime \prime }y_{i}^{\prime \prime }:\;i=1,2,\ldots ,k\right\}$ with $y_{i}^{\prime \prime }=y_{i}$, where each vertex $x_{i}^{\prime \prime }$ of $H_{2}^{*}$ is a leaf. We then obtain $G\wedge {\left\{x_{i}y_{i}\right\}}_{1}^{k}$ to be disconnected and to have two subgraphs $H_{1}^{*}$ and $H_{2}^{*}$. We claim that $\kappa_{d}^{\prime }\left(G\right)\leq \kappa_{d}\left(G\right)$ by the above deduction.

For showing $\kappa_{d}\left(G\right)\leq 2\kappa_{d}^{\prime }\left(G\right)$, we take an edge subset $\left\{e_{1},e_{2},\ldots ,e_{k}\right\}$ of E(G) with $k=\kappa_{d}^{\prime }\left(G\right)$. Notice that the e-divided graph $G\wedge {\left\{e_{i}\right\}}_{1}^{k}$ is obtained by dividing each edge $e_{i}=u_{i}v_{i}$ into two edges, $e_{i}^{\prime }=u_{i}^{\prime }v_{i}^{\prime }$ and $e_{i}^{\prime \prime }=u_{i}^{\prime \prime }v_{i}^{\prime \prime }$. It means that dividing each vertex of the vertex set $X=\{u_{i},v_{i}:\;i=1,2,\ldots ,k\}$ enables us to obtain a v-divided graph G∧X, which is disconnected; immediately, we obtain the inequalities $\kappa_{d}\left(G\right)\leq 2\kappa_{d}^{\prime }\left(G\right)$, as desired. The examples depicted in Figure 3 and Figure 4 are to show the boundaries of this theorem. The proof of the theorem is complete. □

**Remark** **3.** *This theorem provides a method for computing graph connectivity.*

### 3.2. Structures of Graphs Based on the v-Divided Connectivity

Let κ(G)=k for a connected graph *G*, so there are subsets $S_{i}\left(k\right)$ of V(G) for i=1,2,…,M(k) and $|S_{i}\left(k\right)|=k$, such that each disconnected graph $G-S_{i}\left(k\right)$ has its own components $G_{i,1},G_{i,2},\ldots ,G_{i,m_{i}}$ with $m_{i}\geq 2$, where M(k) is the number of subsets of *G*. We have two new parameters:

$$m^{-}\left(k\right)=\min\left\{m_{i}:S_{i}\left(k\right)\subset V\left(G\right),i=1,2,\ldots ,M\left(k\right)\right\},$$

and

$$m^{+}\left(k\right)=\min\left\{m_{i}:S_{i}\left(k\right)\subset V\left(G\right),i=1,2,\ldots ,M\left(k\right)\right\}.$$

We generalize the above two parameters to other disconnected graphs $G-S_{i}\left(r\right)$ for i=1,2,…,M(r) with possible *r* with respect to $k\leq r\leq \kappa_{M}\left(G\right)$. Thereby, we have $m^{-}\left(r\right)$ and $m^{+}\left(r\right)$ with $k\leq r\leq \kappa_{M}\left(G\right)$ having no subset *Y* with $\kappa_{M}\left(G\right)+1$ elements making G−Y disconnected. We have another concept regarding graph connectivity which is $n_{dis}\left(G\right)$ defined by $n_{dis}\left(G\right)=\max\left\{m^{+}\left(r\right):\;k\leq r\leq \kappa_{M}\left(G\right)\right\}$. Thus, we have a subset X⊂V(G) such that the disconnected graph G−X has the maximum number $n_{dis}\left(G\right)$ of components. Hence, G−X can be characterized as follows:

**Theorem** ** 3.** *Suppose that a connected graph G has a subset X holding G−X to be not connected, and $n\left(G-X\right)=n_{dis}\left(G\right)$ if and only if each component of G−X is a complete graph.*

**Proof.**  Let the disconnected graph G−X has its own components $H_{1},H_{2},\ldots ,H_{n}$, where $n=n_{dis}\left(G\right)$. Clearly, all components $H_{j}$ are complete graphs. If some $H_{j}$ has two nonadjacent vertices *u* and *v*, then a subset $X\left(u,v\right)=V\left(H_{j}\right)-\left\{u,v\right\}$ means that $H_{j}-X\left(u,v\right)$ has two isolated vertices *u* and *v*, so $n_{dis}\left(G\right)\geq n+1$, which contradicts $n=n\left(G-X\right)=n_{dis}\left(G\right)$. □

**Remark** **4.** *This theorem provides several perspectives for discussing graph connectivity, such as a half-K-group of v-divided graphs, connected-perfect, and so on.*

Since G−X has the maximum components $H_{1},H_{2},\ldots ,H_{n}$ with $n=n\left(G-X\right)=n_{dis}\left(G\right)$, we have a v-divided graph G∧X with its components $Q_{1},Q_{2},\ldots ,Q_{n}$ holding $V\left(Q_{j}\right)=V\left(H_{j}\right)\cup Y_{j}$ and $E\left(Q_{j}\right)=E\left(H_{j}\right)\cup E_{j}\cup E_{j}^{*}$, where $Y_{j}=X\setminus X_{j}$ and $E_{j}=\left\{xy:\;x\in V\left(H_{j}\right),y\in Y_{j}\right\}$ and $E_{j}^{*}=\left\{uv:\;u,v\in Y_{j}\right\}$, and each vertex of $X_{j}$ is not adjacent with any vertex of $H_{j}$ for j=1,2,…,M(k). Thus, we can coincide these v-divided graphs $Q_{1},Q_{2},\ldots ,Q_{n}$ to obtain the original graph *G* (or other graphs *H* with connectivity κ(H)=k, where *H* differs from *G*). What structure does each $Q_{j}$ have? Here, $Q_{j}=K_{n_{j}}\cup G\left[E_{j}\right]\cup G\left[E_{j}^{*}\right]$, where $n_{j}=\left|V\left(H_{j}\right)\right|$ since $H_{j}$ is a complete graph, and $V\left(G\left[E_{j}\right]\right)\cap V\left(G\left[E_{j}^{*}\right]\right)=Y_{j}$ such that

(a-1) $V\left(K_{n_{j}}\right)\cap V\left(K_{n_{i}}\right)=\emptyset$; (a-2) $V_{j,s}=V\left(G\left[E_{j}^{*}\right]\right)\cap V\left(G\left[E_{s}^{*}\right]\right)\neq \emptyset$ for some j≠s. 

If (a-2) holds true, we can coincide $Q_{j}$ with $Q_{s}$ together by overlapping the same vertices of $V_{j,s}$ in $Q_{j}$ and $Q_{s}$. We call $Q_{1},Q_{2},\ldots ,Q_{n}$ a *half-K-group of v-divided graphs*.

**Figure 5:**
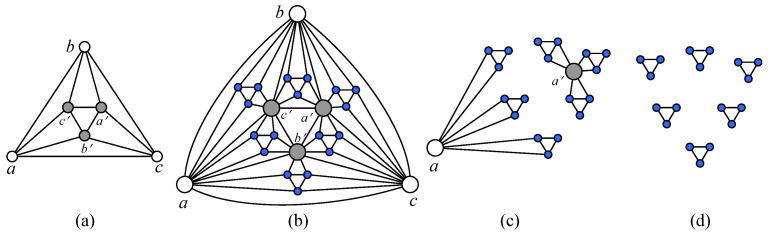
(**a**) A Sierpinski model S(1) has $n_{dis}\left(S\left(1\right)\right)=2$ and three connected-perfect subsets. (**b**) Another Sierpinski model S(2) has $n_{dis}\left(S\left(2\right)\right)=8$ and three connected-perfect subsets $X_{a}=\left\{b,b^{\prime },c,c^{\prime }\right\}$, $X_{b}=\left\{a,a^{\prime },c,c^{\prime }\right\}$ and $X_{c}=\left\{b,b^{\prime },a,a^{\prime }\right\}$. (**c**) S(2) is 4-connected and also v-divided 4-connected, but it is e-divided 2-connected (see Figure 6). (**d**) The disconnected graph $S\left(2\right)-X^{2}$ has $n(S\left(2\right)-X^{2})=6$ components, which is the most, where $X^{2}=\left\{a,a^{\prime },b,b^{\prime },c,c^{\prime }\right\}$ is a connected-perfect subset of S(2).

We consider a subset X⊂V(G) to be *connected-perfect* if $n\left(G-X\right)=n_{dis}\left(G\right)$, and |X|≤|Y| for any subset *Y* holding G−Y to be disconnect and $n\left(G-Y\right)=n_{dis}\left(G\right)$. It may be interesting to find such connected-perfect subsets for a connected graph, and, moreover, whether a connected graph does have a unique connected-perfect subset, and so on. In [14], The Sierpinski model S(t) has its own vertex number $n_{v}^{S}\left(t\right)$ and edge number $n_{e}^{S}\left(t\right)$ as: $n_{v}^{S}\left(t\right)=\frac{3\cdot 6^{t}+12}{5}$ and $n_{e}^{S}\left(t\right)=\frac{9\cdot 6^{t}+6}{5}$ at time step *t*. For instance, the disconnected graph $S\left(t\right)-X^{t}$ has $n\left(S\left(t\right)-X^{t}\right)=6^{t-1}$ components for t≥2, and each $X^{t}$ is a connected-perfect set since $n\left(S\left(t\right)-X^{t}\right)=n_{dis}\left(S\left(t\right)\right)$, as well as $|X^{t}|=3+\frac{3}{5}\left(6^{t-1}-1\right)$. As t=2, the Sierpinski model S(2) is v-divided 4-connected and e-divided 2-connected (see Figure 5) [15].

Thus, we obtain the structure of a connected graph having the most components of a disconnected graph G−X for some subset *X* of a connected graph *G* below.

**Theorem** ** 4.** *A connected graph G holds $n_{dis}\left(G\right)=n\left(G-X\right)=n$ true for some subset X⊂V(G) if and only if there are its subgraphs $Q_{1},Q_{2},\ldots ,Q_{n}$ such that each $Q_{j}-Y_{j}$ with $Y_{j}=V\left(Q_{j}\right)\cap X$ is a complete graph for j=1,2,…,n. In other words, the v-divided graph G∧X has its own components just to be $Q_{1},Q_{2},\ldots ,Q_{n}$.*

**Figure 7:**
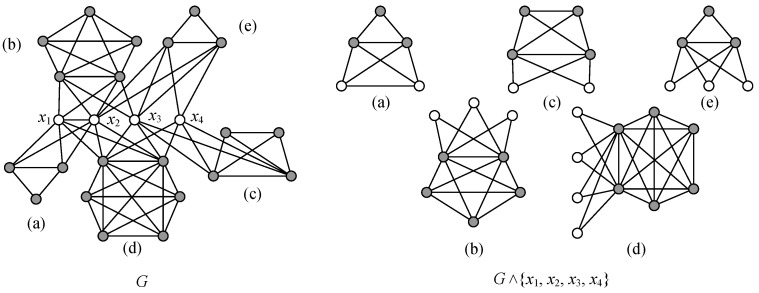
(**a**–**e**) is a connected graph *G* in the left, and (**a**–**e**) is a v-divided graph $H=G\wedge \{x_{1},x_{2},x_{3},x_{4}\}$ in the right.

We show an example in Figure 7 for understanding Theorem 4. Moreover, we can see that $G-\{x_{1},x_{2},x_{3},x_{4}\}$ has five components in Figure 7, namely, $n_{dis}\left(G\right)=5$, and *G* is 2-connected. In fact, *H* can produce two or more graphs *Q* such that $Q-\{x_{1},x_{2},x_{3},x_{4}\}$ has five components, and *Q* is 2-connected. The inverse of Theorem 4 is shown below.

**Theorem** **5.** *Let each connected graph $L_{i}$ be $k_{i}$-connected with $k_{i}\geq k\geq 1$ and i=1,2,…,m. If there exists a nonempty set X holding $V\left(L_{i}\right)\cap V\left(L_{j}\right)=X$ true for i≠j and |X|=k, then the connected graph G obtained by coinciding each vertex of X of $L_{i}$ with its same vertex of X of $L_{j}$ (i≠j) is k-connected. Conversely, the v-divided graph G∧X has its own components $L_{1},L_{2},\ldots ,L_{m}$.*

### 3.3. An Application of the v-Divided and v-Coincident Operations

Coinciding two nonadjacent vertices x,y of a connected graph *G*, if N(x)∩N(y)=∅ until the resultant graph *H* has no two nonadjacent vertices u,v holding N(u)∩N(v)=∅ true, we call *H* an *overlapping kernel graph* of *G*. Evidently, there are two or more such overlapping kernel graphs of *G*. What characteristics does *H* have? First of all, *H* is connected obviously. An Euler’s graph is one without odd-degree vertex, and such graphs were obtained first by the famous mathematician Euler. We present new characters for Euler’s graphs here.

**Theorem** **6.** *A simple graph G of n edges is a connected Euler’s graph if and only if*

*(E-1)*  *It can be divided into a cycle $C_{n}$ by a series of vertex divided operations;*
*(E-2)*  *Its overlapping kernel graph H holds diameter D(H)≤2 and no vertex of H is adjacent to two vertices of odd-degrees in H, simultaneously.*

**Proof.**  We prove (E-1) first.

*Necessary.* Let *G* be a connected Euler’s graph, not being a cycle. A 2-degree 2-connected v-divided operation is defined as follows: Take a vertex $x_{1}$ with its neighbor set $N\left(x_{1}\right)=\left\{y_{1},y_{2},\ldots ,y_{d}\right\}$, where d≥4 is the degree of the vertex *x*. We divide the vertex $x_{1}$ into two vertices, $x_{1}^{\prime }$ and $x_{1}^{\prime \prime }$, such that $N\left(x_{1}^{\prime }\right)=\left\{y_{1},y_{2}\right\}$ and $N\left(x_{1}^{\prime \prime }\right)=N\left(x_{1}\right)\setminus N\left(x_{1}^{\prime }\right)$; the resultant graph is an Euler’s graph still, and is denoted as $G\wedge x_{1}$. If $G\wedge x_{1}$ is disconnected, so $G\wedge x_{1}$ has only two components, $G_{1}$ and $G_{2}$, where $x_{1}^{\prime }\in V\left(G_{1}\right)$ and $x_{1}^{\prime \prime }\in V\left(G_{2}\right)$, then we modify $N\left(x_{1}^{\prime }\right)=\left\{y_{1},y_{3}\right\}$ and $N\left(x_{1}^{\prime \prime }\right)=N\left(x\right)\setminus N\left(x^{\prime }\right)$, since $y_{3}$ is connected with each vertex of $G_{2}$, and $y_{2}$ is connected with each vertex of $G_{1}$. The new graph is connected and denoted by $H_{1}=G\wedge x_{1}$ again. Clearly, $|V\left(G\right)|+1=|V\left(H_{1}\right)|$ and $\left|E\left(G\right)\right|=E\left(H_{1}\right)$. We refer to this procedure of dividing the vertex $x_{1}$ by *2-degree 2-connected v-divided operation*. Thereby, we can perform such operation on $H_{1}$ to obtain a connected Euler’s graph $H_{2}=H_{1}\wedge x_{2}$ holding $|V\left(H_{1}\right)|+1=|V\left(H_{2}\right)|$ and $|E\left(H_{1}\right)|=E\left(H_{2}\right)$ true, if $x_{2}$ has degree ≥4 in $H_{1}$. We continue in this way until we obtain a connected Euler’s graph $H_{m}=H_{m-1}\wedge x_{m}$, in which there is no vertex having degree more than 4. In other words, $H_{m}$ is a cycle.

*Sufficiency.* We can coincide a pair of vertices, $x_{m}^{\prime }$ and $x_{m}^{\prime \prime }$, of the cycle $H_{m}$ for obtaining a connected Euler’s graph $H_{m-1}$ if $N\left(x_{m}^{\prime }\right)\cap N\left(x_{m}^{\prime \prime }\right)=\emptyset$, and then coinciding two vertices $x_{m-1}^{\prime }$ and $x_{m-1}^{\prime \prime }$ of the connected Euler’s graph $H_{m-1}$ produces another connected Euler’s graph $H_{m-2}$ when $N\left(x_{m-1}^{\prime }\right)\cap N\left(x_{m-1}^{\prime \prime }\right)=\emptyset$. Thus, we obtain the original Euler’s graph *G* by performing a series of v-coinciding operations, because each $H_{k}$ is a connected Euler’s graph for i=1,2,…,m.

We come to show (E-2) in the following.

*The proof of “if”.* We perform a so-called *non-neighbor coincident operation* on a connected graph $G_{1}^{*}=G$, and this operation is defined as follows: Coinciding two nonadjacent vertices u,v of $G_{1}^{*}$ if N(u)∩N(v)=∅, here, “nonadjacent vertices u,v” means that the graph $G_{1}^{*}$ contains no edge uv. Thus, we perform such operation on the graph until the last graph $G_{k}^{*}$ has no two nonadjacent vertices x,y, holding N(x)∩N(y)=∅ for some k≥1. $G_{k}^{*}$ is just an *overlapping kernel graph* of the original graph $G_{1}^{*}$. Obviously, $G_{k}^{*}$ has its own diameter $D(G_{k}^{*})\leq 2$, and no vertex of $G_{k}^{*}$ is adjacent to two vertices of odd degrees simultaneously, as if $G_{1}^{*}$ is a connected Euler’s graph.

*The proof of “only if”.* Suppose that the overlapping kernel graph *H* of the connected graph *G* has its own diameter D(H)≤2 and no vertex has two neighbors of odd degrees in *H*. If D(H)=1, *H* is a complete graph, and has no vertex having two neighbors of odd degrees. Thereby, *H* is a connected Euler’s graph. Performing a series of 2-degree 2-connected v-divided operations on *H* produces the original graph *G*. Clearly, *G* is a connected Euler’s graph. If D(H)=2, any pair of nonadjacent vertices u,v of *H* holds N(u)∩N(v)≠∅ true, and *H* is a connected Euler’s graph since *H* has no odd-degree vertex. Obviously, the original graph *G* is the result of v-dividing *H* after performing a series of 2-degree 2-connected v-divided operations.

The proof of the theorem is complete. □

Notice that each Sierpinski model S(t) is a connected Euler’s graph, and it can be v-divided into a cycle $C_{n_{e}\left(t\right)}$ at each time step *t*, where $n_{e}\left(t\right)=\left|E\left(S\left(t\right)\right)\right|=\frac{1}{2}\left(9\cdot 6^{t}+6\right)$ is the edge number of the Sierpinski model S(t) at time step *t*.

## 4. Conclusions

To investigate an open question proposed by Battaglia et al. in [11], we defined two types of divided operations, called the v-divided operation and e-divided operation, respectively, as well as their inverse operations: the v-coincident operation and e-coincident operation. Thereby, we defined the v-divided connectivity $\kappa_{d}$ and the e-divided connectivity $\kappa_{d}^{\prime }$, and showed $\kappa_{d}^{\prime }\leq \kappa_{d}\leq 2\kappa_{d}^{\prime }$ for all simple graphs (respectivenetworks), and $\kappa_{d}$ is equivalent to the traditional vertex connectivity κ [13]. However, finding the v-divided *k*-connectivity for each maximal planar graph of order n≥5 and determining the v-divided *k*-connectivity of an Euler’s graph are not easy.

We consider that finding connected-perfect subsets of a connected graph (respective networks) may be interesting and important for investigating topological structures of GNs. As known, the Sierpinski model S(t) is scale-free, and we discover that each vertex of a connected-perfect subset *X* of S(t) is a scale-free vertex; in other words, *X* controls the topological structure of S(t). Does each connected-perfect subset of a *scale-free deterministic network* control the topological structure of the network?

For a connected simple graph (respective networks) *G* with its *k*-connectivity, our v-divided graph (respective networks) $G\wedge {\left\{x_{i}\right\}}_{1}^{k}$ can reconstruct the original graph (respective networks) *G* easily, but it is very difficult to rebuild *G* from the disconnected vertex-deleting graph (respective networks) $G-{\left\{x_{i}\right\}}_{1}^{k}$, in general. Nevertheless, the structure of the disconnected graph (respective networks) $G-{\left\{x_{i}\right\}}_{1}^{k}$ is unique, rather than $G\wedge {\left\{x_{i}\right\}}_{1}^{k}$ containing components $L_{1},L_{2},\ldots ,L_{m}$ with $2\leq m\leq n(G-{\left\{x_{i}\right\}}_{1}^{k})$, where $n(G-{\left\{x_{i}\right\}}_{1}^{k})$ is the number of components of the disconnected graph (respective networks) $G-{\left\{x_{i}\right\}}_{1}^{k}$. We characterized the disconnected graph G−X obtained by deleting a nonempty subset *X* of the vertex set V(G) from a connected graph *G*, in which n(G−X) is the maximum, and proposed that each component of G−X is a complete graph.

We emphasize that our v-divided operation can dilute a connected Euler’s graph into a cycle; conversely, our e-coincident operation can concentrate a cycle to an Euler’s graph. Moreover, each connected simple graph can be obtained by deleting some edges from some Euler’s graph. We ask the following: How many different Euler’s graphs made by a given cycle are there?

## Figures and Tables

**Figure 6:**
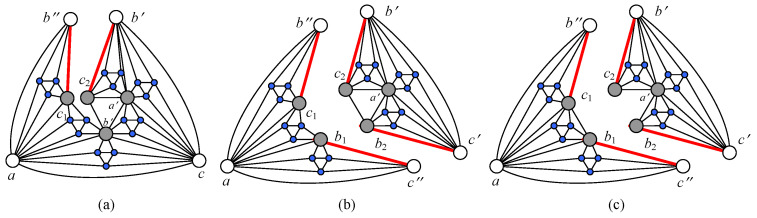
The Sierpinski model S(2) is e-divided 2-connected: (**a**) Dividing an edge $bc^{\prime }$ of the Sierpinski model S(2) into two edges $b^{\prime \prime }c_{1}$ and $b^{\prime }c_{2}$ for obtaining an e-divided graph $S\left(2\right)\wedge bc^{\prime }$. (**b**) Dividing an edge $b^{\prime }c$ of the e-divided graph $S\left(2\right)\wedge bc^{\prime }$ into two edges $b_{1}c^{\prime \prime }$ and $b_{2}c^{\prime }$ for obtaining another e-divided graph $S\left(2\right)\wedge \left\{bc^{\prime },b^{\prime }c\right\}$. (**c**) Another e-divided graph different from the one shown in (**b**).

**Table 1:** The mathematical symbols.

N(x)	The set of all neighbors of a vertex *x* in a simple graph
|N(x)|	The degree of the vertex *x*
δ(H)	The minimum degree
κ(H)	The vertex connectivity
$\kappa^{\prime }\left(H\right)$	The edge connectivity
$\kappa_{d}\left(H\right)$	The v-divided k-connected

## Data Availability

Not applicable.
